# The prevalence and mortality of hyponatremia is seriously underestimated in Chinese general medical patients: an observational retrospective study

**DOI:** 10.1186/s12882-017-0744-x

**Published:** 2017-10-31

**Authors:** Jinling Hao, Yang Li, Xuehan Zhang, Cheng Pang, Yi Wang, Sagar U. Nigwekar, Ling Qiu, Limeng Chen

**Affiliations:** 1 Department of Nephrology, Peking Union Medical College Hospital, Chinese Academy of Medical Sciences & Peking Union Medical College, No. 1, Shuaifuyan, Wangfujing St, Beijing, 100730 China; 2 Health Care Department, Peking Union Medical College Hospital, Chinese Academy of Medical Sciences & Peking Union Medical College, Beijing, 100730 China; 3 Medical Record Department, Peking Union Medical College Hospital, Chinese Academy of Medical Sciences & Peking Union Medical College, Beijing, 100730 China; 4 Department of Laboratory, Peking Union Medical College Hospital, Chinese Academy of Medical Sciences & Peking Union Medical College, No. 1, Shuaifuyan, Wangfujing St, Beijing, 100730 China; 5 Nephrology Division, Massachusetts General Hospital, Boston, MA USA

**Keywords:** Epidemiology, Hospitalization, Hyponatremia, In-hospital mortality

## Abstract

**Background:**

Hyponatremia (serum sodium concentration < 135 mmol/L) is the most common electrolyte abnormality and is a predictor of the mortality of hospitalized patients in Western countries. However, hyponatremia data are lacking in Asian countries. Here we evaluate the epidemiology and mortality of hyponatremia in general medical hospitalized patients in China.

**Methods:**

This is a cohort study of 154,378 adults who were hospitalized between 2008 and 2012 at a teaching hospital in Beijing. We identified hospital patients with hyponatremia and calculated the prevalence and in-hospital mortality of hyponatremia. We also conducted a comprehensive retrospective review of the medical records of patients who had severe hyponatremia (serum sodium <120 mmol/L) during hospitalization in 2012.

**Results:**

The overall prevalence of hyponatremia at some point during hospitalization was 17.5% (26,990 patients), but only 0.26% (394 patients) of cases were identified with the diagnostic code of hyponatremia. Hyponatremia was more common in patients with infectious disease, cancer, or cardiovascular disease as the primary reason for hospitalization based on discharge diagnosis, with prevalences of 33.0, 25.9 and 24.9%, respectively. The in-hospital mortality was 0.48% amongst patients without hyponatremia compared to 3.57 and 20.23% in patients with serum sodium levels of 130–134 and <120 mmol/L, resulting in multivariable adjusted odds ratios (ORs) of 4.8 (95% CI 4.3–5.4) and 32.9 (95% CI 25.2–42.3), respectively. The mortality risk increased with increasing severity of hyponatremia in all diagnostic groups. After the multivariate adjustment, only the Charlson Comorbidity Index and age were independently associated with death risk (OR 1.36, 95% CI 1.14–1.64 and OR 1.04, 95% CI 1.00–1.09, respectively) in the patients with severe hyponatremia.

**Conclusions:**

Hyponatremia is highly prevalent among Chinese hospitalized patients and is associated with increased in-hospital mortality risk. Physicians should raise awareness to improve the prognosis of hyponatremia.

**Electronic supplementary material:**

The online version of this article (10.1186/s12882-017-0744-x) contains supplementary material, which is available to authorized users.

## Background

Hyponatremia (serum sodium level < 135 mmol/L) is the most common electrolyte abnormality in hospitalized patients with the reported prevalence ranging from 5 to almost 35% in Western countries [1–7]. To date there is no study providing the frequency of hyponatremia in patients hospitalized for general medical conditions in China.

Hospitalized patients are subject to multiple stimuli such as pain, illness, severe nausea, and high exposure to medication. Several studies have described an association between hyponatremia and increased mortality rates [5, 8–10]. Whether this is a direct effect of hyponatremia, or whether hyponatremia is simply a marker for underlying disease severity, remains uncertain.

To date, few data exist on the prevalence and prognostic effect of hyponatremia on admitted general medical patients in Asia. In addition, previous studies have not examined differences in the risk of death with hyponatremia in different subpopulations. Using data from a large unselected group of adults who received care at the Peking Union Medical College Hospital (PUMCH, Beijing, China), we performed this retrospective population-based cohort study to investigate the relationship between hospital-associated hyponatremia and in-hospital mortality across the primary diagnostic categories in a broad population.

## Methods

### Study setting and population

We extracted administrative and laboratory data from all hospital admissions to the PUMCH, which is a 1800 bed tertiary teaching hospital that provides care to a socioeconomically diverse population within the northern part of China and the surrounding region. Data on all patients admitted to PUMCH between January 1, 2008 and December 31, 2012 were obtained through the patient record system of PUMCH. The study period was selected based on the availability of complete data in the system. We excluded patients <18 years (*n* = 12,424) and without data of baseline characteristics (*n* = 10,518). Obstetrical admissions (*n* = 19,936) were not analysed because of the expected presence of mild physiologic hyponatremia in pregnancy (Additional file 1: Figure S1). For patients with multiple hospitalizations, we included only the hospitalization with the lowest serum sodium level in the analysis set.

Demographics and International Classification of Disease, Ninth Revision, Clinical Modification (ICD-9-CM) diagnostic codes were obtained from medical records. The discharge abstract file was linked with the hospital’s electronic laboratory database from which we extracted serum Na values for the corresponding hospitalization. The primary analyses examined the lowest level of measured in-patient sodium as the exposure of interest. We used ICD-9-CM codes and diagnosis-related groups to identify medical conditions present during hospitalization.

Based on the primary discharge diagnosis recorded in the discharge record, we categorized patients into 11 major disease groups: cancer, cardiovascular disease, endocrine disease, gastrointestinal disease, infectious disease, muscle and connective tissue disease, neurologic disease, observation for suspected disease, respiratory disease, urogenital disease, and “others”.

We examined a specific cohort of patients in greater detail: those who were admitted from January 1, 2012 to December 31, 2012 with serum sodium levels <120 mmol/L. A comprehensive chart review of these cases was undertaken to determine the clinical course, including the length of hospital stay and serum sodium level at admission, lowest overall serum sodium and serum sodium level at discharge. Comorbidities were quantified using the Charlson Comorbidity Index (CCI), which is not affected by serum sodium levels [11, 12].

### Measurement of serum sodium values

Using a computer retrieval of archived laboratory data, we identified the lowest serum sodium recorded for all participants. Serum sodium was measured using indirect ion-selective electrodes. Sodium levels were corrected for the dilutional effect associated with hyperglycaemia using the following previously validated methods: sodium_corr_ = sodium +1.6 × [(glucose - 100)/100] [13]. We defined normonatremia as serum sodium values between 135 and 145 mmol/L and hyponatremia as serum sodium values below 135 mmol/L. The lowest recorded hyponatremia was further stratified into four categories (<120, 120–124, 125–129 and 130–134 mmol/L) in accordance with previous studies. Severe hyponatremia was defined as serum sodium <120 mmol/L.

### Assessment of mortality

The information on vital status of each inpatient at discharge was obtained from hospital administrative records. The death reason and diagnosis of every dead patient was reviewed. The detailed medical history records and death records were reviewed in patients with serum sodium levels <120 mmol/L from January 1, 2012 to December 31, 2012 in greater detail.

### Statistical analysis

We first examined the distribution of baseline characteristics according to categories of serum sodium levels. Continuous variables were described as the mean ± standard deviation (SD) or median (25-75th percentiles) where appropriate. Categorical variables were expressed using proportions.

Age-specific prevalence and in-hospital mortality were plotted to depict the age effects on the prevalence and in-hospital mortality of patients with hyponatremia. We also reported in-hospital mortality across serum sodium groups. We then proceeded to perform a series of analyses to better understand the relationship between in-hospital mortality and serum sodium level. Crude and adjusted odds ratios (ORs) with 95% confidential intervals (CIs) were estimated for associated factors of in-hospital mortality such as age, gender, and primary diagnosis by using univariate and multivariate logistic regression models.

A logistic regression was used to test univariate associations between the in-hospital mortality and a comprehensive list of baseline demographic and clinical characteristics of patients with severe hyponatremia. Characteristics that were found to be significantly associated with in-hospital mortality were included in subsequent multivariate models.

All statistical analyses were conducted using IBM SPSS Statistics 14.0 (IBM Corporation, Armonk, NY, USA). A two-sided *P*-value <0.05 was considered statistically significant.

## Results

### Prevalence of hyponatremia

**Fig. 1:**
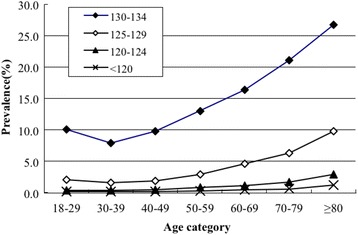
Prevalence of hyponatremia in different age stages

Amongst the 197,256 hospitalizations in the study cohort, a total of 154,378 met our inclusion and exclusion criteria (Additional file 1: Figure S1). The overall prevalence of hyponatremia at some point during hospitalization was 17.5% (26,990 patients), whereas only 0.26% (394 patients) of cases were identified with a diagnostic code of hyponatremia. The proportion of patients in the four hyponatremia categories (130–134, 125–129, 120–124 and <120 mmol/L) was 13.0, 3.3, 0.9 and 0.3%, respectively. Those who had hyponatremia during their hospital stay were significantly older (56.5 vs. 48.8 years; *p* < 0.01) and more frequently males (50.5 vs. 36.1%; *p* < 0.01). The demographic and clinical characteristics of the patients with and without hyponatremia are shown in Table 1. The prevalence of hyponatremia was significantly different in each disease group, which was more common in patients with infectious disease, cancer, or cardiovascular disease as the primary reason for hospitalization based on primary discharge diagnosis. Age stratification revealed the hyponatremia is more prevalent with age, and an increasing trend was significant in patient age groups over 40 years of age (Fig. 1).

**Table 1:** Demographic and clinical characteristics of hospitalized individuals with and without hyponatremia

	Serum sodium concentration (mmol/L)				
	≥135 (*n* = 127,388)	130–134 (*n* = 20,076)	125–129 (*n* = 5035)	120–124 (*n* = 1355)	<120 (*n* = 524)
Age (years)	48.8 ± 15.9	55.6 ± 17.2	58.8 ± 17.3	60.1 ± 16.7	59.9 ± 17.2
Male (%)	36.1	50.0	51.9	52.7	51.7
Primary discharge diagnosis (%)					
Infectious disease	67.0	21.7	8.5	2.1	0.7
Cancer	74.1	18.4	5.4	1.6	0.5
Cardiovascular disease	75.1	19.0	4.7	0.9	0.3
Gastrointestinal disease	77.5	16.9	4.3	0.9	0.4
Observation for suspected disease	78.6	14.0	5.5	1.3	0.6
Muscle and connective tissue disease	78.7	16.9	3.3	0.9	0.3
Respiratory disease	78.8	13.5	5.4	1.6	0.8
Others	84.9	11.7	2.5	0.7	0.3
Neurologic disease	85.8	10.2	2.8	0.9	0.4
Endocrine disease	90.3	6.8	1.7	0.7	0.5
Urogenital disease	93.6	5.2	0.8	0.3	0.1

### In-hospital mortality

**Fig. 2:**
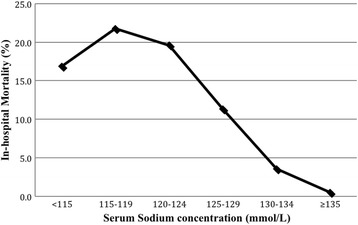
The relationship between in-hospital mortality rate and serum sodium concentration

The overall in-hospital mortality was 6.15% in patients with hyponatremia compared to 0.48% amongst patients without hyponatremia (Table 2). Absolute mortality was increased in all categories of hyponatremia. The mortality tended to increase as the serum sodium level fell from 134 to 115 mmol/L. As the serum sodium level fell below 115 mmol/L, the trend reversed such that mortality amongst the 166 patients with serum sodium <115 mmol/L (16.9%) was lower than the mortality amongst the 358 patients with serum sodium in the 115 to 119 mmol/L range (21.8%) (Fig. 2). Differences in mortality persisted after multivariable adjustment in all categories. The higher mortality risk in patients with hyponatremia of any severity than in non-hyponatremia patients persisted after controlling for age, gender and primary discharge diagnosis, yielding adjusted ORs of 4.8 (95% CI 4.3–5.4), 14.4 (95% CI 12.7–16.3), 28.9 (95% CI 24.2–34.2) and 32.9 (95% CI 25.5–42.3) for sodium levels of 130–134, 125–129, 120–124 and <120 mmol/L, respectively (Table 3).

**Table 2:** Mortality in patients with and without hyponatremia

	Sodium Concentration (mmol/L)					
	≥135 (*n* = 127,388)	<135 (*n* = 26,690)	130–134 (*n* = 20,076)	125–129 (*n* = 5035)	120–124 (*n* = 1355)	<120 (*n* = 524)
In-hospital mortality (%)	0.48	6.15	3.57	11.36	19.63	20.23
Crude	1 (ref)	13.5 (12.3–14.8)	7.6 (6.8–8.5)	26.4 (23.4–29.6)	50.3 (43.0–58.7)	52.2 (41.6–65.5)
Age-adjusted	1 (ref)	9.8 (8.8–10.7)	5.7 (5.1–6.4)	18.0 (16.0–20.4)	34.1 (29.1–40.1)	35.6 (28.1–45.0)
Age, gender-adjusted	1 (ref)	9.6 (8.7–10.5)	5.6 (5.0–6.3)	17.6 (15.6–19.9)	33.4 (28.4–39.2)	34.8 (27.5–44.1)
Multivariable-adjusted	1 (ref)	8.1 (7.4–8.9)	4.8 (4.3–5.4)	14.4 (12.7–16.3)	28.9 (24.4–34.2)	32.9 (25.5–42.4)

**Table 3:** Odds Ratio of in-hospital mortality in selected subgroups of patients with and without hyponatremia

	Serum sodium concentration (mmol/L)				
	≥135	130–134	125–129	120–124	<120
Gender					
Male	1 (ref)	4.0 (3.5–4.6)	10.9 (9.2–12.8)	23.6 (18.9–29.5)	26.3 (18.7–37.0)
Female	1 (ref)	6.0 (5.0–7.1)	20.2 (16.8–24.4)	37.1 (28.6–48.1)	42.4 (28.9–62.0)
Age					
18–29	1 (ref)	6.2 (3.6–10.5)	28.5 (16.6–49.1)	44.3 (18.4–106.7)	69.9 (23.0–212.2)
30–39	1 (ref)	9.0 (5.4–15.0)	41.4 (24.3–70.6)	46.7 (20.3–107.4)	25.7 (5.6–118.0)
40–49	1 (ref)	6.4 (4.4–9.2)	30.6 (20.8–45.0)	63.8 (38.1–106.7)	60.6 (25.5–144.3)
50–59	1 (ref)	7.8 (6.0–10.3)	22.6 (16.4–31.0)	51.9 (34.7–77.7)	60.7 (31.8–115.6)
60–69	1 (ref)	4.9 (3.7–6.4)	16.9 (12.7–22.4)	29.2 (19.5–43.5)	38.8 (22.5–67.0)
70–79	1 (ref)	3.3 (2.7–4.1)	8.4 (6.7–14.1)	23.0 (16.9–31.2)	24.6 (15.4–39.1)
≥ 80	1 (ref)	2.2 (1.7–2.9)	4.3 (3.2–5.8)	6.7 (4.4–10.3)	7.2 (3.0–13.2)
Primary diagnosis					
Infectious disease	1 (ref)	2.8 (1.6–5.0)	5.0 (2.7–9.4)	7.6 (3.2–18.2)	15.4 (4.9–48.9)
Cancer	1 (ref)	6.4 (5.3–7.7)	23.8 (19.5–29.1)	58.9 (45.1–77.0)	55.7 (36.4–85.3)
Cardiovascular disease	1 (ref)	2.3 (1.8–3.0)	6.2 (4.6–8.4)	13.2 (8.3–21.1)	14.6 (6.7–31.8)
Gastrointestinal disease	1 (ref)	5.2 (3.2–8.2)	11.2 (6.5–19.3)	19.5 (9.2–41.3)	45.0 (16.9–119.5)
Observation for suspected disease	1 (ref)	4.6 (2.6–8.2)	12.7 (7.0–23.0)	10.6 (3.8–29.8)	45.9 (16.5–127.9)
Muscle and connective tissue disease	1 (ref)	6.0 (4.0–9.0)	27.8 (17.8–43.4)	29.6 (15.0–58.4)	52.1 (21.4–126.6)
Respiratory disease	1 (ref)	4.0 (2.9–5.5)	6.3 (4.4–8.9)	7.2 (4.3–12.1)	12.2 (6.3–23.6)
Others	1 (ref)	3.9 (2.4–6.3)	12.2 (7.1–21.1)	29.0 (15.3–55.0)	21.9 (7.7–62.3)
Neurologic disease	1 (ref)	5.4 (1.9–15.3)	2.0 (0.2–16.4)	21.4 (5.0–91.6)	
Endocrine disease	1 (ref)	7.1 (2.0–25.6)	25.8 (6.9–96.4)	17.5 (2.0–151.9)	
Urogenital disease	1 (ref)	8.9 (3.9–20.5)	35.3 (14.4–87.1)	166.2 (66.7–413.8)	45.7 (8.9–235.8)

**Fig. 3:**
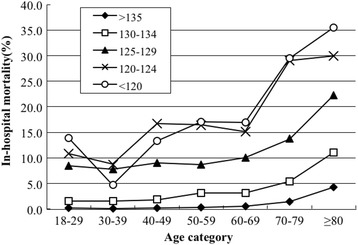
In-hospital mortality rate of hyponatremia patients by age category

The in-hospital mortality rate of patients with hyponatremia was the lowest (2.92%) at ages 30 to 39 years and approached 15.90% in patients over 80 years of age, showing a rapid increase over 70 years of age (Fig. 3). Compared to the 18 to 29 age group, only participants aged 70 to 79 years and ≥80 years had higher in-hospital mortality after controlling for gender and primary discharge diagnosis with ORs of 1.98 (95% CI 1.53–2.57) and 3.70 (95% CI 2.82–4.85), respectively (Additional file 2: Table S1).

### Mortality according to primary discharge diagnostic group

Patients with hyponatremia had increased in-hospital mortality across all major categories of primary discharge diagnoses compared to patients without hyponatremia. The mortality risk increased with increasing hyponatremia severity in all diagnostic groups, and primary discharge diagnoses of cancer (from 6.4 (95% CI 5.3–7.7) for sodium levels of 130–134 mmol/L to 55.7 (95% CI 36.4–85.3) for sodium levels <120 mmol/L), muscle and connective tissue disease (from 6.0 (95% CI 4.0–9.0) for sodium levels of 130–134 mmol/L to 52.1 (95% CI 21.4–126.6) for sodium levels <120 mmol/L) and urogenital disease (from 8.9 (95% CI 3.9–20.5) for sodium levels of 130–134 mmol/L to 45.7 (8.9–235.8) for sodium levels <120 mmol/L) have a higher risk (Table 3).

### Analysis of cases with severe hyponatremia (serum sodium <120 mmol/L)

In the study period, 20.2% (*n* = 124) of patients with severe hyponatremia died during their index admission. There were 135 cases with severe hyponatremia from January 1 to December 31 of 2012, of whom 30 died in the hospital. Table 4 summarizes the demographic and clinical features of these patients. Those who died during their hospital stay were significantly older (65.8 vs. 57.9 years; *p* = 0.020) and had a significantly higher median CCI (4 vs. 1; *p* = 0.001). They also had a significantly lower serum albumin level (28.3 vs. 33.7 g/L; *p* = 0.001).

**Table 4:** Demographic and clinical characteristics of hospitalized individuals with severe hyponatremia (n = 135)

	Dead (*n* = 30)	Alive (*n* = 105)	*P* value
Age (years)	65.8 ± 18.7	57.9 ± 18.0	0.020
Male (%)	43.3	53.5	0.448
Length of hospital stay (days)	12.5 (3.0, 29.5)	14.0 (7.0, 25.5)	0.345
CCI	4 (2, 9)	1 (0, 3)	0.001
CCI category			<0.001
0	2 (6.7%)	32 (30.5%)	
1	5 (16.6%)	26 (24.8%)	
≥ 2	23 (76.7%)	47 (44.8%)	
Age (years)	65.8 ± 18.7	57.9 ± 18.0	0.020
Male (%)	43.3	53.5	0.448
At admission			
Serum albumin (g/L)	28.3 ± 8.1	33.7 ± 7.3	0.001
Serum glucose (mmol/L)	8.3 ± 3.0	6.7 ± 3.0	0.036
Serum creatinine (umol/L)	61.0 (46.0, 116.0)	51.0 (42.0, 72.0)	0.501
Urea (mmol/L)	5.3 (4.1, 14.4)	4.6 (3.4, 6.5)	0.394
Urine acid (mmol/L)	292.5 ± 165.9	199.2 ± 70.4	0.043
Serum sodium level (mmol/L)			
at admission	121.2 ± 11.0	121.7 ± 10.0	0.737
lowest	114.6 ± 5.4	114.3 ± 4.6	0.752
at discharge	132.5 ± 8.5	131.4 ± 7.5	0.820
Community acquired hyponatremia	25 (83.3%)	89 (84.8%)	0.849
Serum sodium back to normal finally	20 (66.7%)	31 (29.5%)	<0.001

*CCI* Charlson Comorbidity Index

We recorded from the discharge abstract the main reason for death as diagnosed by the patient care team. Advanced cancer was reported in 11 participants (36.7%), septic shock in 6 (20.0%), pulmonary infection in 5 (16.7%), haemorrhagic shock in 3 (10.0%), respiratory failure in 3 (10.0%), and cardiac shock in 1 (3.3%). When comparing the patients with serum sodium levels <115 mmol/L and 115 to 119 mmol/L, there was no difference between the in-hospital mortality rate (20.7 vs. 23.3%; *p* = 0.710), CCI (2 vs. 1; *p* = 0.570), and serum albumin level at admission (32.3 vs. 32.6 g/L; *p* = 0.856) (Additional file 3: Table S2).

In the univariate logistic regression analyses, CCI, serum albumin level, serum glucose and calcium level at admission and lowest serum sodium level during the hospital stay had strong relationships with in-hospital mortality, whereas after the multivariate adjustment, only CCI and age were independently associated with death risk (OR 1.36, 95% CI 1.14–1.64 and OR 1.04, 95% CI 1.00–1.09, respectively) (Table 5).

**Table 5:** Multivariate analysis of association of selected factors with mortality in hospitalized individuals with severe hyponatremia (n = 135)

Variable	Mortality OR (95% CI)	*P* value
Age	1.04 (1.00–1.08)	0.050
Admission		
Serum albumin	0.91 (0.83–1.01)	0.065
Serum glucose	1.21 (0.96–1.53)	0.105
Serum calcium	0.27 (0.05–1.54)	0.140
Serum sodium lowest	1.08 (0.93–1.24)	0.325
CCI	1.36 (1.14–1.64)	0.001

*CCI* Charlson Comorbidity Index

In the sensitivity analysis, the results were similar when we analysed the participants whose serum sodium levels were <115 mmol/L (data not shown).

## Discussion

This study represents the largest analysis of hyponatremia amongst unselected hospitalized adults in Asia. The prevalence of hyponatremia in hospitalized individuals is approximately 17.5%. Our estimate is in accordance with the recent large-scale cohort studies reported by Waikar et al. [3] and Holland-Bill et al. [1], in which the serum sodium was measured within 24 h following admission or the first in-patient sodium measurement. As indicated by the divergent results of previous studies, the variability of hyponatremia prevalence amongst the hospitalized population was a result of the use of different cut-off points amongst the examined series [3, 14] and study population compositions [3, 6]. Up to now, there are only several studies providing the frequency of hyponatremia in patients with certain diseases in China. According to published articles, the prevalence of hyponatremia was 9.2% in hospitalized patients with heart failure, 32.7% in patients with central nervous system disease and 33.8% in patients with pneumocardial disease in China [15–17].

Notably, the present study showed that hyponatremia codification in our series is very far from the true prevalence of this condition amongst hospitalized patients. Our results are comparable with a published study from Spain in which only 1.5% of patients were found with a diagnostic code of hyponatremia in a total of 2,134,363 admittances analysed [18]. There may be three reasons for this. First, from most physicians’ perspectives, hyponatremia seems secondary to the patient’s principal condition. Second, physicians appear to have a low awareness of the importance of hyponatremia and its clinical impact. Finally, since the codes assigned at discharge, it is possible that hyponatremia resolved by the time of discharge was not captured in the discharge diagnosis. In one study, Correia et al. demonstrated that in 63 cases of severe hypontremia, the diagnosis of hyponatremia was mentioned in the discharge file of 43 patients (68.25%). If the incidence of hyponatremia had been calculated based only on final diagnosis written in the discharge letter, it would be only 3.96% [19].

Our large study population allowed us to assess the mortality risk associated with different levels of hyponatremia and across numerous diagnostic groups while controlling for important confounders. The in-hospital mortality rate for patients with hyponatremia in our study was 6.15%, which was equivalent to that in previous studies applying the same definition for hyponatremia [1–4]. We observed that the risk of mortality in individuals with hyponatremia is evident even in mild cases (serum sodium concentration = 130–134 mmol/L), which constitutes the majority of cases of hyponatremia. Our results in Chinese hospitalized patients were in similar with those in Western countries, which gave an indication of the significance of abnormal serum sodium during hospitalization in China.

Our study showed that the risk of death in hyponatremia patients was higher for all diagnoses. The significance of hyponatremia varied according to the clinical context. In the stratified analysis, we did find a heightened association between in-hospital mortality and hyponatremia with a primary diagnosis of cancer, muscle and connective tissue disease, and urogenital disease. The prognostic value of hyponatremia for the risk of death has been previously described for cancer [20–23], but not to our knowledge in urogenital disease or muscle and connective tissue disease. Although the exact reason is unclear, we believe that the involvement of multiple organs and complexity of the diseases puts these patients at a higher risk of death. To improve the prognosis of hyponatremia patients, regular monitoring of serum sodium levels is necessary.

A strong association between hyponatremia and increased in-patient mortality has been demonstrated in our study and several other studies [1–7]. However, whether hyponatremia by itself contributes to mortality or merely represents a surrogate marker for the severity of the underlying diseases remains a contentious issue. Some authors have suggested that hyponatremia merely reflects other co-morbidities, i.e., an epiphenomenon of illness [4, 9]. Others have argued that hyponatremia itself contributes independently to in-hospital death and is the probable cause of excess mortality [3, 6]. In our study, the review of patients with severe hyponatremia who died showed that death was mostly attributable to conditions other than hyponatremia. The outcome analysis showed that CCI and age were the only significant predictive risk factors for mortality in severe hyponatremia. These results may challenge the hypothesis that a causal link exists between hyponatremia and mortality. Hyponatremia can be a marker of the severity of an underlying disease. Similar to the results found by Chawla et al., once the serum sodium concentration fell below 120 mmol/L, the mortality rate did not appear to increase as the severity of hyponatremia worsened [4]. Additionally, a recent study found that a decrease in serum sodium below a threshold of 132 mmol/L did not contribute to a further increase in overall mortality risk [1]. Although the dose–response effect of progressive hyponatremia on mortality has been noted in other previous studies [3, 6, 9], the paucity of data regarding the effect of hyponatremia on in-hospital mortality highlights the need for more studies to address this critical question. Another possibility here is a survivorship bias-a phenomenon commonly referred to as “depletion of susceptibles”, i.e., those remaining in the cohort are likely resistant to the effect of hyponatremia on mortality.

This study has several strengths. We studied a large and unselected population with an array of comorbid conditions. This is the first study, to our knowledge, to provide insights into the seriously underestimated prevalence of patients with hyponatremia in the Asian population. Our large study population allowed us to examine the mortality risk associated with different levels of hyponatremia and across numerous diagnostic groups. There are also several important limitations to consider. We relied on ICD-9 codes to classify patients in subgroups and for use in multivariable models. The accuracy of ICD-9 codes for diagnoses, such as syndromes of inappropriate antidiuretic hormone and volume depletion, has not been studied. The extent of misclassification due to the inaccuracy of the ICD-9 code is not known and may have affected our estimate of mortality within clinical subgroups. Secondly, it is impractical to define causes for hyponatremia when performing a large-scale epidemiological study such as ours. Another limitation was our inability to measure the severity of illness during hospitalization. There are no data on the mortality after hospital discharge and, hence, we were restricted to evaluating in-hospital mortality. The relationship between hyponatremia and mortality has previously been shown to persist after discharge [3]. Finally, our study is a cross-sectional cohort study with a single serum sodium measurement that does not reflect changes in clinical management.

## Conclusions

In conclusion, we identified that the prevalence and mortality of hyponatremia were seriously underestimated in the hospitalized Chinese population. Increased awareness and regular monitoring of the serum sodium levels in patients with cancer, muscle and connective disease, or urogenital disease, who were first demonstrated to have a higher risk of death, are necessary.

## Additional files

Additional file 1: Figure S1. Flowchart used to define the study cohort. (DOC 45 kb)

Additional file 2: Table S1. Multivariable logistic regression analysis of in-hospital mortality for patients with hyponatremia. (DOC 40 kb)

Additional file 3: Table S2. Compare of demographic and clinical characteristics of hospitalized individuals with serum sodium (*n* = 135). (DOC 35 kb)

## Abbreviations

CCI: Charlson Comorbidity Index
ICD-9-CM: International Classification of Disease, Ninth Revision, Clinical Modification
