# Supplementary material for: The prevalence and mortality of hyponatremia is seriously underestimated in Chinese general medical patients: an observational retrospective study
Source: BMC Nephrol. 2017 Oct 31;18:328. doi: 10.1186/s12882-017-0744-x (PMC5664828; doi:10.1186/s12882-017-0744-x)
Supplement: Supplementary file 1 — Flowchart used to define the study cohort. (DOC 45 kb) [file 12882_2017_744_MOESM1_ESM.doc]

**Figure S1. Flowchart used to define the study cohort**

197,256 hospitalized patients in PUMCH between January 01, 2008 to December 31, 2012

Excluded hospitalized patients with missing data of baseline characteristics (n=10,518)

Excluded patients less than 18 years old in admission date (n=12,424)

Excluded hospitalized patients with pregnancy (n=19,936)

174,314 adult hospitalized patients

154,378 adult hospitalized patients
