# Supplementary material for: The prevalence and mortality of hyponatremia is seriously underestimated in Chinese general medical patients: an observational retrospective study
Source: BMC Nephrol. 2017 Oct 31;18:328. doi: 10.1186/s12882-017-0744-x (PMC5664828; doi:10.1186/s12882-017-0744-x)
Supplement: Supplementary file 2 — Multivariable logistic regression analysis of in-hospital mortality for patients with hyponatremia. (DOC 40 kb) [file 12882_2017_744_MOESM2_ESM.doc]

**Table S1. Multivariable logistic regression analysis of in-hospital mortality for patients with hyponatremia**

| **Parameter** | **Total**  **(n)** | **Death**  **(n)** | **Mortality Rate (%)** | **Univariate** | **Multivariate** |
| --- | --- | --- | --- | --- | --- |
| All patients | 26990 | 1661 | 6.15% |  |  |
| Sex |  |  |  |  |  |
| Female | 13360 | 734 | 5.49% | 1 (Ref) | 1 (Ref) |
| Male | 13630 | 927 | 6.80% | 1.24 (1.13-1.36) | 0.98 (0.89-1.09) |
| Age |  |  |  |  |  |
| 18-29 | 2386 | 75 | 3.14% | 1 (Ref) | 1 (Ref) |
| 30-39 | 2569 | 75 | 2.92% | 0.93 (0.67-1.28) | 0.91 (0.65-1.26) |
| 40-49 | 4109 | 153 | 3.72% | 1.19 (0.90-1.58) | 1.03 (0.78-1.38) |
| 50-59 | 5397 | 273 | 5.06% | 1.64 (1.23-2.13) | 1.26 (0.96-1.64) |
| 60-69 | 5311 | 292 | 5.50% | 1.79 (1.38-2.32) | 1.27 (0.97-1.66) |
| 70-79 | 5205 | 473 | 9.09% | 3.08 (2.40-3.94) | 1.98 (1.53-2.57) |
| ≥80 | 2013 | 320 | 15.90% | 5.82 (4.50-7.55) | 3.70 (2.82-4.85) |

Adjusted for age, sex and primary discharge diagnosis
