# Supplementary material for: The prevalence and mortality of hyponatremia is seriously underestimated in Chinese general medical patients: an observational retrospective study
Source: BMC Nephrol. 2017 Oct 31;18:328. doi: 10.1186/s12882-017-0744-x (PMC5664828; doi:10.1186/s12882-017-0744-x)
Supplement: Supplementary file 3 — Compare of demographic and clinical characteristics of hospitalized individuals with serum sodium (n = 135). (DOC 35 kb) [file 12882_2017_744_MOESM3_ESM.doc]

**Table S2. Compare of demographic and clinical characteristics of hospitalized individuals with serum sodium (n=135)**

| Variable | Serum Sodium level (mmol/L) | | P value |
| --- | --- | --- | --- |
|  | <115 (n=58) | 115-119 (n=77) |  |
| Age | 63.8 ± 16.7 | 56.6 ± 19.1 | 0.022 |
| Admission |  |  |  |
| albumin | 32.3 ± 7.7 | 32.6 ± 8.0 | 0.856 |
| Serum creatinine | 51.0 (44.0, 69.0) | 55.0 (42.0, 76.5) | 0.329 |
| Serum glucose | 7.6 ± 4.1 | 6.7 ± 3.1 | 0.182 |
| Serum sodium |  |  |  |
| level at admission | 117.5 ± 10.8 | 124.7 ± 8.6 | <0.001 |
| hyponatremia at admission | 51 (87.9%) | 63 (81.8%) | 0.332 |
| back to normal finally | 15 (25.9%) | 36 (46.8%) | 0.013 |
| CCI | 2 (1, 5) | 1 (0, 4) | 0.570 |
| Mortality | 12 (20.7%) | 18 (23.3%) | 0.710 |

CCI: Charlson Comorbidity Index
